# Functional and Social Vulnerability in Patients With Chronic Obstructive Pulmonary Disease (COPD) Managed in Primary Care: A Population-Based Cohort Study

**DOI:** 10.7759/cureus.114975

**Published:** 2026-08-22

**Authors:** Josep Montserrat-Capdevila

**Affiliations:** 1 Teaching Unit of Family and Community Care, Primary Care and Community Health, Catalan Health Institute (ICS), Lleida, ESP; 2 Faculty of Medicine, University of Lleida, Lleida, ESP

**Keywords:** copd, copd (chronic obstructive pulmonary disease), frailty, morbidity and mortality, primary medical care, social vulnerability

## Abstract

Background: Patients with chronic obstructive pulmonary disease (COPD) are increasingly recognized as a heterogeneous population in whom prognosis depends not only on respiratory impairment but also on functional decline, multimorbidity, and social vulnerability. However, the prognostic value of routinely recorded functional and social vulnerability markers in primary care has been insufficiently investigated.

Objective: The objective of the study is to determine whether routinely recorded functional and social vulnerability markers are independently associated with all-cause mortality among patients with spirometry-confirmed COPD managed in primary care.

Methods: We conducted a population-based retrospective cohort study using electronic health records from primary care centers in Catalonia, Spain. Adults with spirometry-confirmed COPD were followed from 2019 to 2023. Functional and social vulnerability markers included living alone, home-care program (ATDOM) enrollment, functional dependency, nursing home residence, dementia, and long-term respiratory support. Multivariable logistic regression was performed to identify independent predictors of all-cause mortality.

Results: Among 2,056 patients, 558 (27.1%) died during follow-up. Patients who died were older, predominantly male, and had a greater burden of comorbidity and functional vulnerability. After adjustment, home-care program enrollment, functional dependency, and nursing home residence remained independently associated with mortality, together with age, male sex, smoking, previous exacerbations, heart failure, atrial fibrillation, chronic kidney disease, and active malignancy. Physical activity and higher FEV₁ were independently associated with lower mortality.

Conclusions: Routinely recorded functional and social vulnerability markers independently identified patients with COPD at increased risk of death beyond traditional clinical predictors. Their incorporation into routine primary care assessment may improve risk stratification and facilitate earlier identification of high-risk patients for personalized multidisciplinary care.

## Introduction

Chronic obstructive pulmonary disease (COPD) is one of the leading causes of morbidity and mortality worldwide and represents a major challenge for primary care. Current international guidelines emphasize that COPD should not be assessed solely according to the degree of airflow limitation but through a multidimensional approach that integrates symptoms, exacerbation history, comorbidities, and treatable traits. Accordingly, COPD prognosis is increasingly recognized as the result of complex interactions between respiratory impairment, systemic disease burden, functional status, and social circumstances rather than lung function alone [1,2].

Several multidimensional prognostic models have been developed to improve risk stratification in COPD. The BODE index demonstrated that combining body mass index, airflow obstruction, dyspnea, and exercise capacity predicts mortality more accurately than forced expiratory volume in one second (FEV₁) alone [3]. Other studies have shown that COPD prognosis depends on multiple clinical factors beyond FEV₁, including exacerbation burden, cardiovascular disease, smoking, physical inactivity, and multimorbidity [4-6]. However, many of these prognostic models require structured clinical assessments, exercise testing, or specialized measurements that are not routinely available in primary care, where most patients with COPD receive long-term follow-up.

Beyond physiological impairment, increasing attention has focused on the impact of functional decline and social vulnerability on health outcomes in chronic diseases. Functional dependency, cognitive impairment, institutionalization, and the need for home-care services reflect cumulative biological and social vulnerability and may identify patients at particularly high risk of adverse outcomes. Although frailty has consistently been associated with mortality in COPD, most published studies have relied on formal frailty scales or comprehensive geriatric assessments that are rarely incorporated into routine primary care practice [7,8]. Social determinants of health also play an important role in COPD outcomes [9]. Previous studies have also demonstrated that survival depends on multiple clinical factors beyond airflow limitation alone [10]. Physical activity has also been consistently associated with improved prognosis and lower mortality in COPD [11]. More recently, the concept of early disease recognition and pre-COPD has broadened the understanding of disease progression and prognosis [12].

Primary care electronic health records routinely capture several indicators of functional and social vulnerability, including living alone, enrollment in the home-care program (ATDOM), functional dependency, nursing home residence, dementia, and long-term respiratory support. These variables may represent pragmatic markers of vulnerability that complement traditional clinical predictors such as lung function, exacerbation history, and comorbidity burden. Nevertheless, their independent prognostic value has been scarcely investigated in large population-based cohorts of patients with spirometry-confirmed COPD managed in primary care.

Because these variables are routinely available without requiring additional assessments, they could provide a simple and readily implementable strategy for improving prognostic stratification in routine clinical practice. Early identification of patients with increased functional or social vulnerability may facilitate proactive multidisciplinary interventions, optimize healthcare resource allocation, and support personalized management strategies.

We hypothesized that routinely recorded functional and social vulnerability markers provide prognostic information beyond established clinical predictors of COPD mortality. Therefore, the aim of this study was to determine the independent association between routinely recorded functional and social vulnerability markers (living alone, enrollment in the home-care program (ATDOM), functional dependency, long-term respiratory support, nursing home residence, and dementia) and all-cause mortality in a population-based cohort of patients with spirometry-confirmed COPD managed in primary care.

## Materials and methods

Study design and setting

We conducted a retrospective population-based cohort study using routinely collected electronic health records from the Primary Care Information System (eCAP) of the Catalan Health Institute (Institut Català de la Salut (ICS)), the largest public primary healthcare provider in Catalonia, Spain. The study included patients receiving routine care at primary care centers in the Lleida Health Region between January 1, 2019, and December 31, 2023.

The eCAP electronic health record prospectively captures demographic characteristics, diagnoses, prescriptions, spirometry, preventive interventions, healthcare utilization, and long-term clinical outcomes as part of routine clinical practice, providing a standardized source of real-world data for epidemiological research.

The study was designed and reported according to the Strengthening the Reporting of Observational Studies in Epidemiology (STROBE) guidelines.

Study population

Eligible participants were adults (≥18 years) with a recorded diagnosis of COPD in the electronic primary care medical record (eCAP) of the Catalan Health Institute (ICS) at baseline and spirometric confirmation of COPD within the previous two years. COPD was defined by persistent airflow obstruction (post-bronchodilator FEV₁/FVC <0.70) documented in the electronic health record. The inclusion and exclusion criteria applied to the study population are summarized in Table 1.

**Table 1:** Inclusion and exclusion criteria. FEV₁, forced expiratory volume in one second; FVC, forced vital capacity

Criterion type	Criterion
Inclusion	Adults aged ≥18 years.
Inclusion	Recorded diagnosis of chronic obstructive pulmonary disease (COPD) in the electronic primary care medical record (eCAP) at baseline.
Inclusion	Available spirometry performed within the previous two years confirming persistent airflow obstruction compatible with COPD (post-bronchodilator FEV₁/FVC <0.70).
Exclusion	Patients without a recorded diagnosis of COPD in eCAP at baseline or without spirometric confirmation of COPD within the previous two years.

The index date corresponded to cohort entry, when baseline demographic, clinical, functional, and social variables were recorded. Participants were followed until death or administrative censoring on December 31, 2023.

A total of 2,056 patients met the eligibility criteria and comprised the final analytical cohort.

Variables

Baseline demographic variables included age and sex. Lifestyle variables comprised smoking status and physical activity, routinely recorded during primary care visits according to standardized electronic health record categories.

Clinical variables included systolic and diastolic blood pressure, body mass index, cardiovascular risk estimated using the REGICOR score, influenza and pneumococcal vaccination status, and spirometric parameters (FEV₁, FVC, and FEV₁/FVC ratio).

The burden of COPD exacerbations during the year preceding cohort entry was identified from routinely collected, pseudonymized healthcare data. Moderate exacerbations were defined as episodes requiring treatment with systemic corticosteroids and/or antibiotics, whereas severe exacerbations were defined as episodes requiring hospital admission with acute exacerbation of COPD recorded as the principal discharge diagnosis. Hospital-based exacerbations were ascertained through the hospital discharge administrative database of the reference hospital. These data were integrated with the primary care electronic health record and included as a marker of previous disease activity.

Baseline comorbidities were ascertained from coded diagnoses recorded in the eCAP electronic health record at cohort entry and included hypertension, dyslipidemia, type 2 diabetes mellitus, chronic kidney disease, atrial fibrillation, heart failure, previous stroke, ischemic heart disease, pulmonary hypertension, bronchiectasis, obstructive sleep apnea, active malignancy, dementia, and alcohol misuse.

The primary exposure variables were six functional and social vulnerability markers routinely recorded as structured variables or coded diagnoses in the eCAP electronic health record: living alone, enrollment in the ATDOM, functional dependency, long-term respiratory support, nursing home residence, and dementia.

Outcome

The primary outcome was all-cause mortality during follow-up, identified through routinely updated electronic health records.

Statistical analysis

Continuous variables are presented as mean (standard deviation) or median (interquartile range), according to data distribution. Categorical variables are presented as frequencies and percentages.

Baseline characteristics were compared between survivors and non-survivors using Student's t-test or the Mann-Whitney U test for continuous variables and the chi-square test or Fisher's exact test for categorical variables, as appropriate. For chi-square tests, the test statistic, degrees of freedom, and Cramér's V as a measure of effect size were reported.

Variables considered clinically relevant a priori, together with those associated with all-cause mortality in the univariable analysis at p<0.05, were entered as candidate covariates into the multivariable logistic regression model. Clinical relevance was considered independently of univariable statistical significance to avoid excluding potentially important confounding or prognostic factors. Odds ratios (ORs) and 95% confidence intervals (95% CIs) were calculated. A reduced multivariable model was subsequently derived from the initial model using backward elimination of non-significant covariates, while retaining clinically relevant variables considered essential for adjustment. The reduced model was intended to provide a parsimonious set of independently associated predictors while preserving clinically meaningful adjustment. Multivariable analyses were performed using complete cases for the variables included in each model, and no imputation of missing data was performed.

All statistical tests were two-sided, and a p-value <0.05 was considered statistically significant. Statistical analyses were performed using R software (R Foundation for Statistical Computing, Vienna, Austria).

Ethical considerations

The study was conducted in accordance with the principles of the Declaration of Helsinki and was approved by the Clinical Research Ethics Committee of IDIAP Jordi Gol (protocol 23/280-P). Because of the retrospective observational design and the use of anonymized routinely collected clinical data, the requirement for informed consent was waived by the Ethics Committee.

## Results

Baseline characteristics

A total of 2,056 patients with spirometry-confirmed COPD were included in the study. During follow-up, 558 patients (27.1%) died, and 1,498 (72.9%) remained alive.

Baseline characteristics according to survival status are presented in Table 2. Patients who died were older (median age, 77.0 vs. 69.0 years; p<0.001) and were more frequently male (85.3% vs. 75.8%; χ²(1)=21.54, p<0.001, Cramér's V=0.102). Significant differences were also observed for smoking status, influenza and pneumococcal vaccination, physical activity, previous-year exacerbations, systolic and diastolic blood pressure, cardiovascular risk estimated using the REGICOR score, and spirometric parameters, including FEV₁/FVC ratio, FEV₁, and FVC (all p<0.05).

**Table 2:** Baseline characteristics of patients with spirometry-confirmed COPD according to all-cause mortality χ², chi-square statistic; df, degrees of freedom; V, Cramér's V; IQR, interquartile range; FEV₁, forced expiratory volume in one second; FVC, forced vital capacity. Test statistics, degrees of freedom, and Cramér's V are reported for categorical variables analyzed using Pearson's chi-square test. Dashes indicate that the statistic or effect size is not applicable. *The p-value for current smoking corresponds to the overall comparison of smoking status (never smoker, current smoker, and former smoker); therefore, a separate chi-square statistic and Cramér's V are not reported for the current-smoker category.

Characteristic	Survivors (n=1,498)	Non-survivors (n=558)	Test statistic (df)	P-value	Effect size (Cramér’s V)
Demographic characteristics					
Male sex, n (%)	1,136 (75.8)	476 (85.3)	χ²=21.54 (1)	<0.001	0.102
Age, years, median (IQR)	69.0 (61.0–76.0)	77.0 (70.2–84.0)	—	<0.001	—
Lifestyle factors					
Current smoker, n (%)	344 (23.2)	100 (18.2)	—*	0.001*	—*
Physically active, n (%)	1,061 (76.7)	270 (61.2)	χ²=89.69 (1)	<0.001	0.209
Clinical variables					
Previous-year exacerbations, median (IQR)	0 (0–0)	0 (0–0)	—	<0.001	—
Systolic blood pressure, mmHg	132 (123–140)	127 (114–139)	—	<0.001	—
Diastolic blood pressure, mmHg	76 (70–83)	71 (62–79)	—	<0.001	—
Body mass index, kg/m²	27.6 (24.5–30.7)	27.3 (24.1–30.3)	—	0.151	—
REGICOR score	5.43 (3.51–8.59)	7.40 (4.05–10.00)	—	0.017	—
Pulmonary function					
FEV₁/FVC	0.63 (0.56–0.67)	0.59 (0.51–0.65)	—	<0.001	—
FEV₁, L	1.71 (1.30–2.18)	1.34 (0.96–1.74)	—	<0.001	—
FVC, L	2.84 (2.25–3.53)	2.39 (1.87–3.02)	—	<0.001	—
Major comorbidities					
Hypertension	942 (62.9)	396 (71.0)	χ²=11.69 (1)	0.001	0.075
Diabetes mellitus	411 (27.4)	206 (36.9)	χ²=17.40 (1)	<0.001	0.092
Dyslipidemia	756 (50.5)	233 (41.8)	χ²=12.36 (1)	0.001	0.078
Active malignancy	304 (20.3)	245 (43.9)	χ²=115.82 (1)	<0.001	0.237
Chronic kidney disease	227 (15.2)	181 (32.4)	χ²=76.35 (1)	<0.001	0.193
Atrial fibrillation	180 (12.0)	175 (31.4)	χ²=106.52 (1)	<0.001	0.228
Heart failure	129 (8.6)	156 (28.0)	χ²=127.43 (1)	<0.001	0.249
Previous stroke	202 (13.5)	145 (26.0)	χ²=45.29 (1)	<0.001	0.148

Regarding comorbidities, mortality was significantly more frequent among patients with hypertension, type 2 diabetes mellitus, active malignancy, chronic kidney disease, atrial fibrillation, previous stroke, ischemic heart disease, heart failure, pulmonary hypertension, and dementia. Conversely, dyslipidemia and prediabetes were more prevalent among survivors.

Functional and social vulnerability markers

All predefined functional and social vulnerability markers were significantly associated with all-cause mortality in the univariable analysis (Table 3). Compared with survivors, patients who died more frequently lived alone (13.8% vs. 6.0%; χ²(1)=33.07, p<0.001, Cramér's V=0.127), were enrolled in the ATDOM (24.4% vs. 4.6%; χ²(1)=176.96, p<0.001, Cramér's V=0.293), had functional dependency (28.5% vs. 10.1%; χ²(1)=107.67, p<0.001, Cramér's V=0.229), resided in nursing homes (16.5% vs. 3.0%; χ²(1)=118.84, p<0.001, Cramér's V=0.240), and had dementia (15.6% vs. 5.9%; χ²(1)=49.29, p<0.001, Cramér's V=0.155). Long-term respiratory support was also more frequent among non-survivors (0.9% vs. 0.2%; Fisher's exact test, p=0.038).

**Table 3:** Functional and social vulnerability markers according to all-cause mortality χ², chi-square statistic; df, degrees of freedom; V, Cramér's V; ATDOM, home-care programme. Test statistics, degrees of freedom, and Cramér's V are reported for variables analyzed using Pearson's chi-square test. Fisher's exact test was used for long-term respiratory support because of small expected cell counts. A dash indicates that the effect size was not reported for Fisher's exact test.

Variable	Survivors (n=1,498)	Non-survivors (n=558)	Test statistic (df)	P-value	Effect size (Cramér’s V)
Living alone	90 (6.0)	77 (13.8)	χ²=33.07 (1)	<0.001	0.127
Home-care programme (ATDOM)	69 (4.6)	136 (24.4)	χ²=176.96 (1)	<0.001	0.293
Functional dependency	151 (10.1)	159 (28.5)	χ²=107.67 (1)	<0.001	0.229
Long-term respiratory support	3 (0.2)	5 (0.9)	Fisher’s exact test	0.038	—
Nursing home residence	45 (3.0)	92 (16.5)	χ²=118.84 (1)	<0.001	0.24
Dementia	88 (5.9)	87 (15.6)	χ²=49.29 (1)	<0.001	0.155

Multivariable analysis

In the multivariable logistic regression model, male sex (OR 2.60, 95% CI 1.74-3.93), increasing age (OR 1.05 per year, 95% CI 1.03-1.07), current smoking (OR 2.51, 95% CI 1.61-3.93), and a higher number of COPD exacerbations during the previous year (OR 1.46, 95% CI 1.16-1.87) were independently associated with increased all-cause mortality.

Active malignancy, chronic kidney disease, atrial fibrillation, heart failure, enrollment in the ATDOM, functional dependency, and nursing home residence also remained independently associated with mortality after multivariable adjustment.

Conversely, sufficient physical activity (OR 0.62, 95% CI 0.46-0.84), higher systolic blood pressure (OR 0.98, 95% CI 0.97-0.99), and better lung function measured by FEV₁ (OR 0.48, 95% CI 0.36-0.63) were independently associated with a lower risk of death.

Living alone and dementia were significantly associated with mortality in the univariable analysis but did not retain statistical significance after multivariable adjustment.

**Figure 1:**
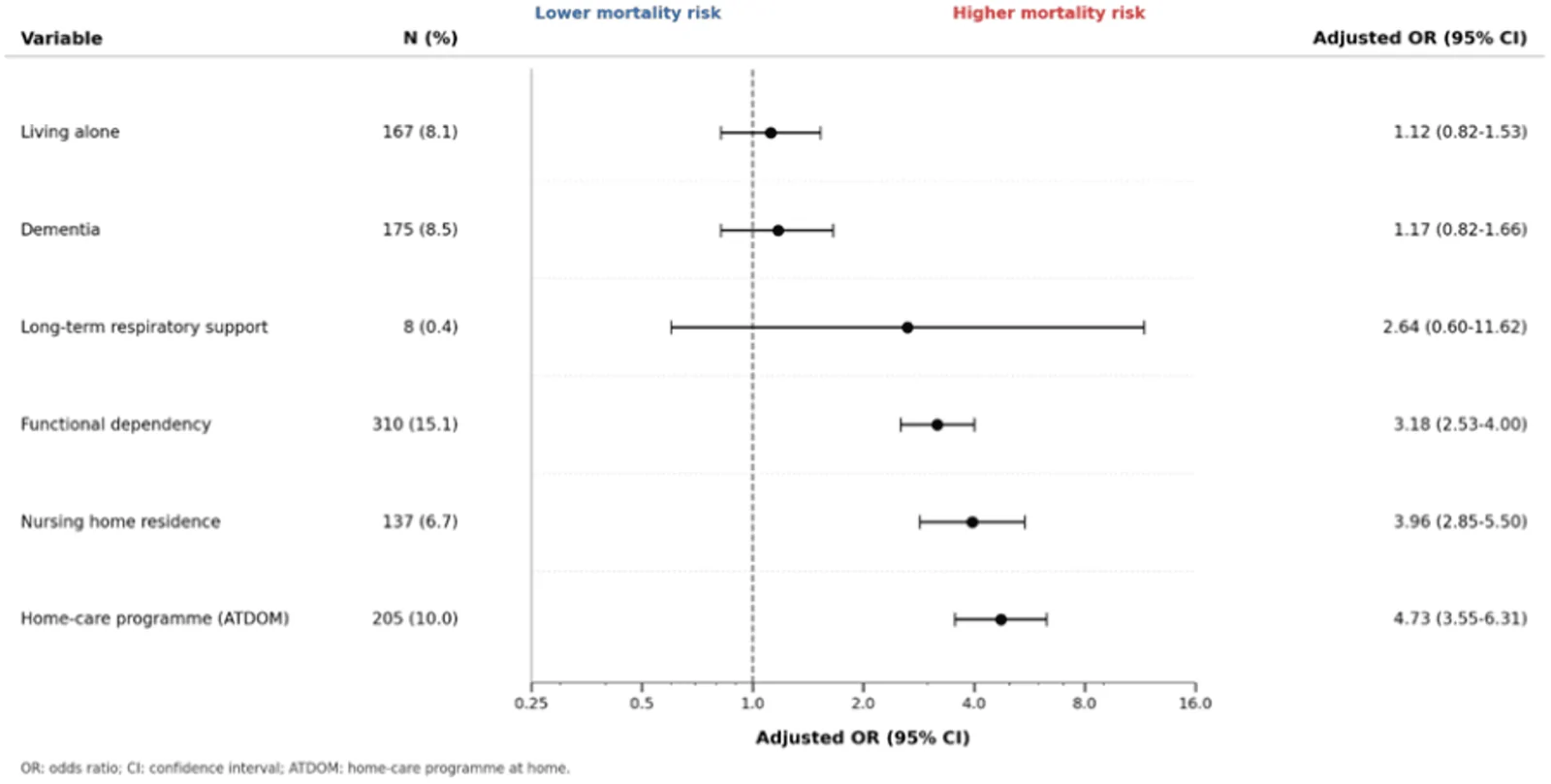
Forest plot of the adjusted associations between functional and social vulnerability markers and all-cause mortality. Adjusted odds ratios and 95% confidence intervals were obtained from the multivariable logistic regression model. Home-care programme enrollment (ATDOM), functional dependency, and nursing home residence remained independently associated with mortality. Living alone, dementia, and long-term respiratory support did not retain statistical significance after adjustment.

The adjusted associations between functional and social vulnerability markers and all-cause mortality are summarized in Figure 1.

## Discussion

Principal findings

In this population-based cohort of patients with spirometry-confirmed COPD managed in primary care, all-cause mortality was substantial, affecting more than one in four patients during follow-up. The main finding of this study is that functional and social vulnerability markers routinely recorded in primary care electronic health records were strongly associated with mortality. Patients who died were more likely to live alone, be enrolled in the ATDOM, have functional dependency, reside in a nursing home, have dementia, or require long-term respiratory support. Importantly, markers reflecting loss of autonomy and healthcare dependency, particularly home-care program enrollment, functional dependency, and nursing home residence, remained independently associated with mortality after adjustment for established clinical predictors.

These findings support a multidimensional understanding of COPD prognosis. Previous studies have demonstrated that survival in COPD depends on multiple clinical and functional factors beyond airflow limitation alone [10]. Current evidence further recognizes that prognosis results from the interaction between respiratory impairment, exacerbation burden, comorbidity, functional reserve, and broader vulnerability [13,14]. Our results extend this concept to primary care by demonstrating that simple functional and social variables routinely available in electronic health records can identify patients at particularly high risk.

Comparison with previous literature

Frailty and functional decline are increasingly recognized as important dimensions of chronic respiratory disease. The European Respiratory Society statement on frailty in chronic lung disease defines frailty as a multidimensional state of reduced physiological reserve that increases susceptibility to adverse outcomes and highlights its role as a potentially treatable trait [13]. Recent systematic reviews have also shown that frailty is common in COPD and is associated with impaired lung function, greater dyspnea, reduced exercise capacity, poorer quality of life, increased hospitalization, and higher mortality [14,15]. Our findings are consistent with these observations while providing a pragmatic primary care perspective: rather than requiring formal frailty instruments, the variables evaluated in this study are already routinely recorded during clinical care.

ATDOM enrollment, functional dependency, and nursing home residence may be considered pragmatic markers of advanced vulnerability. These variables likely reflect multiple overlapping domains, including reduced mobility, diminished physiological reserve, multimorbidity, cognitive impairment, the need for professional support, and limited capacity for self-management. Their association with mortality is therefore clinically plausible. In everyday primary care, these markers are easier to identify and operationalize than formal frailty scales while still providing clinically meaningful prognostic information.

The social dimension of COPD has received less attention than physiological impairment or multimorbidity. Nevertheless, social determinants of health are increasingly recognized as important contributors to health outcomes in chronic diseases [9]. Emerging evidence also indicates that social frailty has important clinical consequences. Hirai et al. reported that social frailty was associated with severe exacerbations and unexpected hospitalization in patients with COPD [16]. In our cohort, living alone was associated with mortality in univariable analysis but lost significance after multivariable adjustment. This suggests that living alone may primarily reflect underlying vulnerability mediated through age, multimorbidity, dependency, or healthcare needs rather than acting as an independent prognostic factor.

Similarly, dementia showed a strong univariable association with mortality but was no longer independently associated after adjustment. This finding suggests that the functional consequences of cognitive impairment may be more prognostically relevant than the diagnosis itself. Once dependency, institutionalization, and comorbidity are considered, dementia appears to contribute limited additional prognostic information, reinforcing the importance of evaluating functional status rather than diagnostic labels alone.

Our results also support the evolving concept that COPD should be understood across a continuum of disease progression rather than solely established airflow obstruction [12]. In addition, previous population-based studies have highlighted the impact of multimorbidity on COPD prognosis, showing that mortality increases substantially with increasing comorbidity burden [17]. In our cohort, active malignancy, chronic kidney disease, atrial fibrillation, and heart failure remained independent predictors of mortality, emphasizing the systemic nature of COPD and the importance of comprehensive patient assessment, consistent with previous population-based evidence [18].

Finally, physical activity was independently associated with lower mortality. This finding is consistent with previous evidence identifying physical activity as an important predictor of survival in COPD [11] and with more recent population-based studies demonstrating that moderate-to-vigorous physical activity is associated with improved survival [19]. Physical activity likely reflects not only lifestyle but also overall physiological reserve, muscle function, cardiometabolic health, autonomy, and social participation. These findings are also consistent with the long-term population-based study by Garcia-Aymerich et al. demonstrating reduced hospital admission and mortality among physically active patients with COPD [20].

Implications for general practice

These findings have direct implications for primary care. Risk stratification in COPD does not necessarily require new or complex assessment tools. General practitioners already have access to clinically meaningful prognostic information through routinely recorded electronic health records and longitudinal knowledge of their patients. Home-care program enrollment, functional dependency, and nursing home residence are simple markers that can help identify patients at high risk of adverse outcomes.

Patients presenting these markers may benefit from proactive multidisciplinary management, including closer follow-up, optimization of inhaler technique, medication review, smoking cessation support, vaccination assessment, individualized exacerbation action plans, pulmonary rehabilitation or physical activity promotion when appropriate, and coordination with nursing staff, caregivers, and social services.

At the population level, these routinely recorded vulnerability markers could also facilitate risk stratification, anticipatory care planning, home-based interventions, and integrated health and social care strategies, particularly in aging populations with increasing multimorbidity and functional dependency.

Strengths and limitations

The strengths of this study include its population-based design, large sample size, inclusion of patients with spirometry-confirmed COPD, and exclusive primary care setting. Unlike hospital-based cohorts, this study reflects the broad spectrum of patients managed in routine primary care, including older adults with multimorbidity, functional decline, and social complexity. The use of routinely recorded electronic health record variables further enhances the clinical applicability of the findings.

Several limitations should be acknowledged. First, the retrospective observational design precludes causal inference, and residual confounding cannot be excluded. Second, functional and social vulnerability markers were derived from routinely collected clinical data rather than validated frailty or social assessment instruments. However, this also represents a pragmatic strength because these variables are readily available without additional assessment. Third, living alone does not adequately capture loneliness, caregiver availability, social support, or socioeconomic deprivation. Fourth, detailed information regarding treatment exposure, inhaler adherence, pulmonary rehabilitation, oxygen therapy, and some markers of disease severity was unavailable. Finally, some vulnerability markers, particularly long-term respiratory support, were infrequent, limiting the precision of their estimates. In addition, the use of routinely collected electronic health record data may introduce selection and information bias because the completeness and accuracy of recorded diagnoses, functional characteristics, and social variables depend on routine clinical documentation; therefore, some degree of misclassification cannot be excluded. Electronic cigarette (EC) use and cumulative vaping exposure were not recorded in the available electronic health records and therefore could not be characterized in this study. Although EC vaping has not been established as a cause of COPD, the absence of information on vaping status, frequency, duration, and cumulative exposure (e.g., puff-years) represents a limitation of the available exposure data. Finally, logistic regression assessed the association between baseline characteristics and mortality status over the study period but did not explicitly account for individual time-to-event or censoring. Accordingly, the reported odds ratios should be interpreted as associations with mortality over the observed follow-up period rather than as estimates of time-dependent mortality risk.

## Conclusions

Routinely recorded functional and social vulnerability markers were independently associated with all-cause mortality in patients with spirometry-confirmed COPD managed in primary care. In particular, ATDOM enrollment, functional dependency, and nursing home residence provided prognostic information beyond traditional clinical risk factors, including age, smoking status, exacerbation history, and lung function.

These findings support a broader, patient-centered approach to COPD management that considers functional status and social vulnerability alongside established clinical risk factors. Because these variables are already available in electronic health records, they may have potential utility for identifying patients at increased risk without requiring additional assessment tools. However, this potential clinical utility should be considered hypothesis-generating. Future prospective studies should determine whether incorporating these markers into routine clinical practice improves risk stratification, clinical decision-making, and patient outcomes.
